# First Dentition

**Published:** 1861-02

**Authors:** 


First Dentition.—May 11.—Case 2.—A negro child, aged 8 months; affected with diarrhea from teething. Diarrhea, properly speaking, is not the name of a disease, but the term by which one of the enteric diseases is known.

The period of first dentition is that fraught with more danger than any other, in the life of children; particularly when it occurs during the warm season.

The danger is still farther increased by epidemic influence, tending to the production of enteric disease in whole neighborhoods, which often exists.

At this tender age the system is naturally impressible, and any source of irritation so disturbs the functions of the nervous system that irritability of the more excitable tissues is induced, which require only a slight cause to establish in them positive inflammation. The alimentary mucous surface is very susceptible to such disturbance from the irritation produced in the gum by a protruding tooth. In addition to this, various forms of functional derangement are set up by reflex nervous action or sympathy, taking their origin from these local points of irritation or inflammation. The brain itself is not unfrequently the subject of functional derangement, the symptoms of which become so prominent, that they are frequently mistaken for those of meningitis, effusion, &c.

The indications in the treatment of such cases are: firstly, to remove the primary source of irritation, by a free incision of the gum; secondly, to allay the enteric irritation, which is more readily met by such means as will quiet general nervous irritability, restrain as much as possible the frequency of the evacuations, and soothe and protect the tender surface from the contact of irritating substances in the canal—these means are opiates, astringents, and demulcents.—*Atlanta Med. and Surg. Journal*

